# Withaferin A Induces Proteasome Inhibition, Endoplasmic Reticulum Stress, the Heat Shock Response and Acquisition of Thermotolerance

**DOI:** 10.1371/journal.pone.0050547

**Published:** 2012-11-30

**Authors:** Saad Khan, Ashley W. Rammeloo, John J. Heikkila

**Affiliations:** Department of Biology, University of Waterloo, Waterloo, Ontario, Canada; Virginia Commonwealth University, United States of America

## Abstract

In the present study, withaferin A (WA), a steroidal lactone with anti-inflammatory and anti-tumor properties, inhibited proteasome activity and induced endoplasmic reticulum (ER) and cytoplasmic HSP accumulation in Xenopus laevis A6 kidney epithelial cells. Proteasomal inhibition by WA was indicated by an accumulation of ubiquitinated protein and a decrease in chymotrypsin-like activity. Additionally, immunoblot analysis revealed that treatment of cells with WA induced the accumulation of HSPs including ER chaperones, BiP and GRP94, as well as cytoplasmic/nuclear HSPs, HSP70 and HSP30. Furthermore, WA-induced an increase in the relative levels of the protein kinase, Akt, while the levels of actin were unchanged compared to control. Northern blot experiments determined that WA induced an accumulation in *bip*, *hsp70* and *hsp30* mRNA but not *eIF-1α* mRNA. Interestingly, WA acted synergistically with mild heat shock to enhance HSP70 and HSP30 accumulation to a greater extent than the sum of both stressors individually. This latter phenomenon was not observed with BiP or GRP94. Immunocytochemical analysis indicated that WA-induced BiP accumulation occurred mainly in the perinuclear region in a punctate pattern, while HSP30 accumulation occurred primarily in a granular pattern in the cytoplasm with some staining in the nucleus. Prolonged exposure to WA resulted in disorganization of the F-actin cytoskeleton as well as the production of relatively large HSP30 staining structures that co-localized with F-actin. Finally, prior exposure of cells to WA treatment, which induced the accumulation of HSPs conferred a state of thermal protection since it protected the F-actin cytoskeleton against a subsequent cytotoxic thermal challenge.

## Introduction

Traditional Indian medicine has utilized plants and their derivatives to treat ailments of the endocrine, cardiopulmonary, and central nervous systems [1], [2]. Known for its anti-inflammatory and cardioactive properties, Ashwaganda (Withania somnifera) has gained more attention lately with its acceptance as a dietary supplement in North America [3]. Of the 40 compounds extracted from the leaves and roots of Ashwaganda, withaferin A (WA; 4β, 27-dihydroxy-1-oxo-5 β, 6 β, epoxy with 2–24 dienolide), a steroidal lactone, is thought to be the active component responsible for its therapeutic properties [2], [4], [5]. For example, WA suppressed Cystic Fibrosis-related inflammation in an in vitro model system by inhibition of the transcription factor, NFkappaB [5]. Furthermore, WA inhibited tumor growth in mice and increased tumor-free survival in a dose-dependent manner and was capable of inducing apoptosis in leukemic cells with no toxicity to normal human progenitor cells [4], [6].

Recently it was reported that WA has an inhibitory effect on ubiquitin-proteasome system (UPS) activity in human prostate cancer cells [3]. The ATP-dependent UPS is responsible for the hydrolysis of most cellular protein and is required for numerous cellular or organismal processes including differentiation, cell cycle progression, apoptosis and development [7]–[9]. A deficiency in the UPS may be involved in the progress of a number of human diseases including Parkinson’s, Alzheimer’s and Huntington’s [10]–[13]. Additionally, proteasome inhibition was reported to induce the accumulation of sets of molecular chaperones collectively termed heat shock proteins (HSPs), in various eukaryotic model systems [7], [14]–[18]. Given that WA induced proteasome inhibition, it was possible that this agent could also induce HSP accumulation. However, very little information is available except for studies that reported WA-induced accumulation of HSP70 in pancreatic cancer and mouse embryo fibroblast cells [19], [20]. Additionally, WA was reported to bind to HSP90 and inhibit its chaperone activity [20]. In a recent study examining the effect of over 80,000 natural and synthetic compounds on a mammalian reporter cell line containing a minimal heat shock element promoter fused to a green fluorescent protein gene, it was determined that WA was a strong inducer of the heat shock response and capable of inhibiting tumour activity in cultured cells and in mice [21].

Vertebrate HSPs consist of several families including HSP90, HSP70 and the small HSPs (sHSPs) [22]–[25]. Two members of the HSP70 family include stress-inducible HSP70 and the resident endoplasmic reticulum (ER) family member called immunoglobulin-binding protein (BiP; also called glucose-regulatory protein 78 or GRP78). Both HSP70 family members act as molecular chaperones by binding to nascent or denatured protein and maintaining them in a folding competent state. The HSP90 family consists of cytoplasmic/nuclear HSP90 and the ER glucose-regulated protein 94 (GRP94). HSP90 assists in protein folding and stabilization while GRP94 plays critical roles in folding client protein and secretory pathways in the ER. The sHSPs consist of ATP-independent molecular chaperones that can bind to unfolded client protein, inhibit their aggregation and maintain them in a soluble and folding competent state such that they can be refolded to their native conformation by other molecular chaperones [25]–[30]. The sHSPs range in size from 16–42 kDa and are quite divergent except for a conserved α-crystallin domain. Additionally, they can form highly multimeric structures that are essential for their chaperone activity. Various studies have shown that sHSPs can function in the acquisition of thermotolerance, actin capping/decapping activity and cellular differentiation. Interestingly, the mutation or accumulation of sHSPs has been associated with various diseases including cataracts, muscle myopathy and Alzheimer’s [31]–[33].

The regulation of stress-induced *hsp90, hsp70* and *shsp* gene expression occurs mainly at the transcriptional level and involves the activation of heat shock factor 1 (HSF1) and its binding to the *cis*-acting DNA sequence, the heat shock element (HSE), located in the upstream regulatory regions of *hsp* genes [23], [34]. Normally, HSF1 preexists in the cell as an inactive monomer bound to HSP90. In response to heat or chemical stress, the accumulation of unfolded, misfolded or damaged protein recruits HSP90 allowing HSF1 to trimerize, become hyperphosphorylated and enable its binding to the HSE to facilitate *hsp* gene transcription [34], [35]. The enhanced expression of *bip* and *grp94* genes is regulated by the unfolded protein response (UPR) [36]. An increase in the level of stress-induced misfolded protein in the ER sequesters BiP from three ER transmembrane transducers leading to their activation. One of the transmembrane proteins is ATF6α that dimerizes and translocates to the Golgi after dissociation from BiP and undergoes proteolytic cleavage to produce a 50 kDa N-terminal protein. This cleaved protein then migrates to the nucleus to activate transcription of *bip* and *grp94* genes by binding to the activating transcription factor (ATF)/cAMP response element (CRE) and ER stress response element (ERSE).

Since *Xenopus* has been used as a research model system for many decades, extensive information is available about its biology at both the cellular and molecular level and the information collected is applicable to human cells. Previous studies in our laboratory have characterized the expression of both ER and cytoplasmic/nuclear HSPs in response to various stresses [25]. For example exposure of *Xenopus laevis* A6 kidney epithelial cells to ER stressors such as A23187 and tunicamycin enhanced the accumulation of BiP and *bip* mRNA [37]. Treatment of *Xenopus* A6 cells with heat shock or chemical stressors were shown to induce the accumulation of HSPs including HSP70 and HSP30, a member of the sHSP family [38]–[41]. Furthermore, HSF1 activation was involved in *Xenopus hsp* gene expression as determined by HSF1-HSE binding experiments and in studies employing an HSF1 inhibitor [40], [42], [43]. Immunocytochemical analysis revealed that HSP30 was localized primarily in the cytoplasm with enrichment in the perinuclear region. Furthermore, HSP30 functions as a molecular chaperone since it inhibited stress-induced aggregation of client protein and maintained them in a soluble and folding-competent state [44]–[47]. Recently, we reported that treatment of A6 cells with proteasome inhibitors induced HSP30 and HSP70 accumulation [18], [48], [49].

As mentioned previously, a few studies have shown that WA can activate the heat shock response. However, only one recent study has reported an effect of this agent on the accumulation of the ER chaperone, BiP [50]. The present study has investigated the effect of WA on both the unfolded protein and heat shock response in *Xenopus laevis* A6 cells. In this study, we have shown that WA treatment inhibits proteasome activity and upregulates the expression of ER (BiP and GRP94) and cytoplasmic/nuclear chaperones (HSP70 and HSP30). Additionally, mild heat treatment in combination with WA resulted in an enhanced accumulation of HSP70 and HSP30 compared to either stressor individually. Immunocytochemical analysis demonstrated that WA treatment conferred thermotolerance enabling the cells to withstand a higher temperature that normally causes F-actin cytoskeletal collapse and cell death.

## Materials and Methods

### Cell Culture and Treatments

*Xenopus laevis* A6 kidney epithelial cells (CCL-102) were purchased from American Type Culture Collection (Rockville, MD) and grown at 22°C in T75 cm^2^ BD Falcon tissue culture flasks (VWR International, Mississauga, ON) employing 55% Leibovitz (L)-15 media supplemented with 10% (v/v) fetal bovine serum, 100 U/mL penicillin and 100 µg/mL streptomycin (all from Sigma-Aldrich, Oakville, ON). Flasks of cells were treated with 2–5 µM withaferin A (≥99% purity; Enzo Life Sciences, Plymouth Meeting, PA; dissolved in DMSO; Sigma), 7 µM A23187 for 24 h at 22°C (dissolved in dimethyl sulfoxide (DMSO); Sigma-Aldrich) or 30 µM MG132 (carbobenzoxy-L-leucyl-L-leucyl-L-leucinal dissolved in DMSO; Sigma-Aldrich; [18]) for periods of time ranging from 4 to 24 h at 22°C. In some experiments WA-treated flasks of cells were heat shocked in a water bath set at 30 or 37°C. After the different treatments, media was removed and 2 mL of 65% HBSS was added to rinse the cells. The cells were then detached from flasks by scraping, suspended in 1 mL of 100% HBSS and transferred to a 1.5 ml microcentrifuge tube. The cell suspensions were centrifuged for 1 min at 21,920 x g and the resultant pellets were stored at −80°C until RNA or protein isolation.

### Detection of Proteasome Chymotrypsin-like Activity

Chymotrypsin-like activity in WA, A23187 and MG132-treated cells was determined using a Promega Proteasome-Glo cell-based luminescent assay (Promega Corp., Madison, WI, USA) following the manufacturer’s protocols. Following WA, A23187 or MG132 treatments, cells were trypsinized and washed in 55% L-15 media. Aliquots of 15,000 cells (counted using a Bright-Line haemocytometer; Hausser Scientific, Horsham, PA, USA), in 100 µL of 55% L-15 media were used for the determination of chymotypsin-like activity. A Victor^3^ luminometer (Perkin Elmer Inc., Waltham, MA, USA) was used to measure the luminescence of each sample.

### RNA Isolation and Northern Blot Analysis

Total RNA was isolated from frozen A6 cell pellets using the QIAgen RNeasy Mini Kit (Qiagen, Mississauga, ON) as detailed in the manufacturer’s instructions. Assessment of RNA quantity and integrity were determined using spectrophotometry employing the NanoDrop ND-1000 (NanoDrop, Waltham, Mass.) and by means of electrophoresis with ethidium bromide staining. Total RNA (10 µg) was subjected to formaldehyde/agarose gel electrophoresis and northern blotting procedures and preparation of digoxigen-labeled antisense *hsp30*, *hsp70*, *bip* and *eIf1α* probes were carried out as previously described [18], [51]–[53]. The RNA blot pre-hybridization, hybridization and washing procedures were performed as outlined in Lang et al. [52]. Chemiluminescent detection was carried out using the manufacturer’s instructions (Roche Diagnostics, Mississauga, ON) and images were visualized by means of a DNR chemiluminescent imager (DNR Bio-Imaging Systems Ltd., QC). The experiments were executed at least three times and densitometric analysis within the range of linearity employed the NIH ImageJ (Version 1.45) software. Average densitometric values were expressed as a percentage of the maximum band and graphed with standard error of the mean represented as vertical error bars. The level of significance of the differences between samples was calculated by one-way ANOVA with a Tukey’s post-test.

### Protein Isolation and Immunoblot Analysis

Total protein was isolated from pelleted A6 cells and subjected to immunoblot analysis as described previously [54]. Immunodetection was carried out using either the polyclonal rabbit anti-BiP (Sigma-Aldrich, 1∶1000 dilution), the monoclonal rat anti-GRP94 (1∶1000 dilution), polyclonal rabbit anti-*Xenopus* HSP30 ([44], 1∶500 dilution), the polyclonal rabbit anti-*Xenopus* HSP70 ([55], 1∶350 dilution), the polyclonal rabbit anti-actin (Sigma-Aldrich, 1∶200 dilution), the polyclonal rabbit anti-Akt (also known as protein kinase B; New England Biolabs, Pickering ON, 1∶1000 dilution) or the monoclonal mouse anti-ubiquitin antibody (Invitrogen, 1∶150 dilution). The nitrocellulose membranes were incubated with alkaline phosphatase-conjugated goat anti-rabbit (BioRad, 1∶3000 dilution), alkaline phosphatase-conjugated goat anti-rat (Sigma-Aldrich, 1∶3000) or the alkaline phosphatase-conjugated goat anti-mouse (BioRad, 1∶1000) secondary antibody. For detection, the membrane was incubated in alkaline phosphatase buffer (100 mM Tris base, 100 mM NaCl, 50 mM MgCl_2_ (pH 9.5)), 0.3% 4-nitro blue tetrazolium (NBT; Roche), and 0.17% 5-bromo-4-chloro-3-indolyl phosphate, toluidine salt (BCIP; Roche) until the bands were visible. The experiments were repeated in triplicate and densitometric analysis in the range of linearity were performed using NIH Image J (Version 1.45) software on all blots as described previously [48]. The average densitometric values were expressed as a percentage of the maximum band for HSP30 or HSP70 and as fold induction compared to control for BiP and GRP94. The normalized data were graphed with standard error of them mean as represented by vertical error bars. The level of significance of the differences between samples was assessed by one-way ANOVA with a Tukey’s post-test.

### Immunocytochemistry and Laser Scanning Confocal Microscopy

Immunofluorescence analysis was carried out as previously described [54]. Briefly, cells were grown at 22°C for 24 h on glass coverslips in petri dishes. Cells were treated with 7 µM A23187 or 30 µM MG132 for 24 h or 5 µM WA for 18 h directly in the petri dishes. For thermotolerance studies some WA-treated cells were further incubated at 37°C for 1 h followed by a recovery at 22°C for 6 h. In these experiments, the petri dishes were wrapped with parafilm, sealed in a plastic bag and then placed in a heated water bath. After treatment, the L-15 media was removed and the cells were washed twice in phosphate-buffered saline (PBS; 1.37 M NaCl, 67 mM Na_2_HPO_4_, 26 mM KCl, 14.7 mM H_2_PO_4_, 1 mM CaCl_2_, 0.5 mM MgCl_2_, pH 7.4) and the coverslips were transferred to new small petri dishes. Following these treatments, cells were rinsed with PBS and fixed in 3.7% paraformaldehyde (BDH, Toronto, ON) for 10 min and then rinsed three times with PBS. The cells were permeabilized with 0.3% Triton X-100 (Sigma-Aldrich) in PBS for 10 min followed by 3 washes with PBS for 5 min each. Cells were then incubated with 3.7% bovine serum albumin (BSA; Fisher Scientific, Ottawa, ON) for 1 h at 22°C or overnight at 4°C. Cells were subsequently incubated with affinity-purified rabbit anti-*Xenopus* HSP30 antibody (1∶500) or polyclonal rabbit anti-BiP antibody (1∶500) in 3.7% BSA for 1 h. After three washes for 3 min each in PBS, cells were indirectly labeled with a fluorescent-conjugated secondary antibody, goat anti-rabbit Alexa Fluor 488 (Molecular Probes, Eugene, OR), at 1∶2000 in 3.7% BSA for 30 min in the dark. F-actin was detected with rhodamine-tetramethylrhodamine-5-isothiocyanate phalloidin conjugated TRITC (Molecular Probes) at 1∶60 in PBS for 15 min in the dark followed by three PBS washes for 5 min each. The coverslips were mounted on glass slides with VectaShield mounting media containing the nucleic acid stain, 4,6-diamidino-2-phenylindole (DAPI; Vector Laboratories Inc., Burlingame, CA), which permits visualization of nuclei. Coverslips were attached to slides using clear nail polish followed by storage at 4°C. Slides were examined by laser scanning confocal microscopy using a Zeiss Axiovert 200 microscope and ZEN 2009 software (Carl Zeiss Canada Ltd., Mississauga, ON).

## Results

### Enhanced Ubiquitinated Protein Accumulation and Inhibition of Proteasomal Chymotrypsin-like Activity in WA-treated Cells

**Figure 1:**
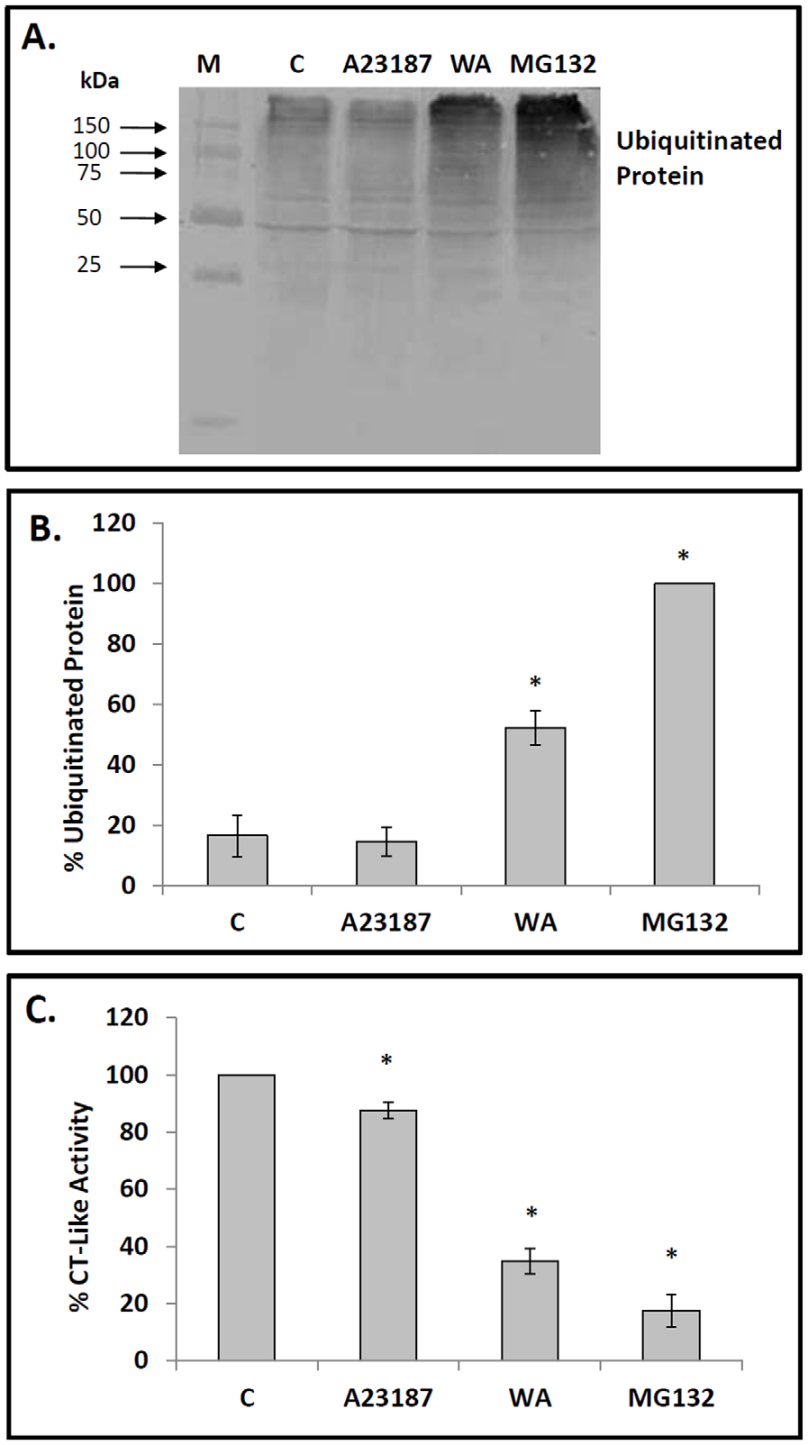
Effect of WA on relative levels of ubiquitinated protein and proteasomal chymotrypsin-like activity. A) Cells were treated with either 5 µM withaferin A (WA) for 16 h or with 7 µM A23187, 30 µM MG132 or the appropriate volume of the DMSO vehicle (C) for 24 h at 22°C. Isolated protein was subjected to immunoblot analysis employing a mouse anti-ubiquitin monoclonal antibody as described in Materials and methods. The positions of molecular mass standards in kDa are shown in the first lane (M). B) Image J software was used to perform densitometric analysis of the signal intensity for ubiquitinated protein bands of western blot images as described in Materials and methods. The data are expressed as a percentage of the lane having maximum density (MG132) while the standard error is represented by vertical error bars. The level of significance of the differences between samples was calculated by one-way ANOVA with a Tukey’s post-test. Significant differences between the control cells and A6 cells treated with 5 µM WA or 30 µM MG132 are indicated as * (*p*<0.05). These data are representative of at least three separate experiments. C) Effect of WA on proteasomal chymotrypsin (CT)-like activity of A6 cells. Cells were treated with WA, A23187 or MG132 as indicated above. A cell-based proteasomal CT-like assay was used to monitor the proteolytic activity as described in Materials and methods. The CT-like activity was measured and expressed as a percentage of the CT-like activity observed in control cells. The level of significance of the differences between samples was calculated by one-way ANOVA with a Tukey’s post-test. Significant differences between control cells and cells treated with WA, A23187 or MG132 are indicated as * (*p*<0.05). These data are representative of three separate experiments.

The effect of WA on proteasome activity in A6 cells was evaluated by measuring the relative levels of ubiquitinated protein and proteasomal chymotrypsin (CT)-like enzyme activity. Immunoblot analysis revealed that the relative levels of ubiquitinated protein in cells treated with 5 µM WA for 16 h or 30 µM MG132, a well-characterized proteasomal inhibitor, for 24 h at 22°C were 5- to 6-fold higher than observed in control cells (Fig. 1A, B). However, treatment of cells with 7 µM A23187, a calcium ionophore and activator of the unfolded protein response, did not result in a significant increase in ubiquitinated protein levels. Furthermore, treatment of cells with higher concentrations of A23187 (10 to 14 µM) did not result in enhanced accumulation of ubiquitinated protein relative to control (data not shown). Analysis of proteasomal CT-like activity revealed that cells treated with WA had a 66% decrease in enzyme activity compared to control (Fig. 1C). The values obtained for cells treated with A23187 or MG132 were 15 and 82%, respectively. These combined results suggest that WA is an inhibitor of proteasome activity in *Xenopus* cultured cells.

### WA Induction of *bip*, *hsp70* and *hsp30* mRNA Accumulation

**Figure 2:**
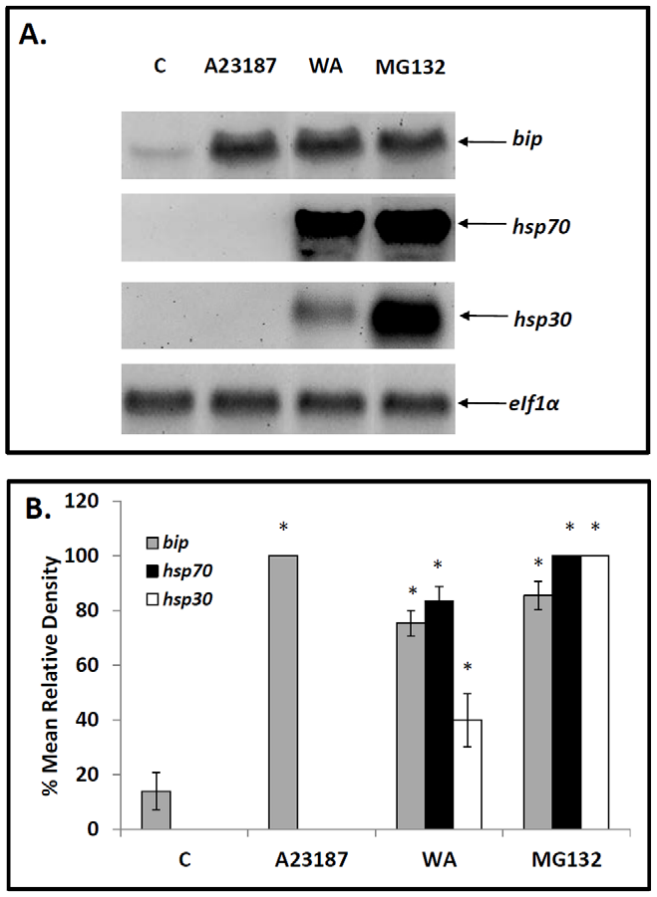
Effect of WA on the relative levels of *bip*, *hsp70, hsp30* and *eIF1α* mRNA. A) Cells were treated with either 5 µM WA for 16 h or 7 µM A23187, 30 µM MG132 or the appropriate volume of the DMSO vehicle (C) for 24 h at 22°C. Cells were harvested and total RNA was isolated. Total RNA (10 µg) was analyzed by northern hybridization analysis using *bip*, *hsp70, hsp30* and *eIF1α* antisense riboprobes as described in Materials and methods. B) Image J software was used to perform densitometric analysis of the signal intensity for *bip* (grey), *hsp70* (black) and *hsp30* (white) mRNA levels of northern blot images as described in Materials and methods. The data are expressed as a percentage of the maximum signal (30 µM MG132 for *hsp70* and *hsp30* mRNA and 7 µM A23187 for *bip* mRNA). The standard error is represented by vertical error bars. The level of significance of the differences between samples was calculated by one-way ANOVA with a Tukey’s post-test. Significant differences between the control cells and treated cells are indicated as * (p<0.05). These data are representative of three separate experiments.

Northern blot analysis was employed to determine the effect of 5 µM WA on the relative levels of the stress-inducible mRNAs encoding BiP, HSP70 and HSP30. As shown in Fig. 2A, enhanced levels of *bip* mRNA were observed in cells treated with WA as well as with the proteasomal inducer, MG132, and the ER stressor, A23187, compared to control cells. Densitometric analysis revealed a 5-, 7- and 6-fold increase in *bip* mRNA levels in cells treated with WA, A23187 and MG132, respectively (Fig. 2B). Unfortunately, GRP94 mRNA levels were not examined since a *Xenopus grp94* cDNA was not available in order to generate an antisense riboprobe. Increased accumulation of *hsp30* and *hsp70* mRNA relative to control was observed in cells treated with WA or MG132 but not with A23187 (Fig. 2A). The relative levels of *eIf1α* mRNA remained unchanged throughout the course of these treatments. Thus, WA treatment of A6 cells induces the accumulation mRNAs encoding both cytosolic and ER HSPs.

### WA Induced Accumulation of BiP, GRP94, HSP70 and HSP30

**Figure 3:**
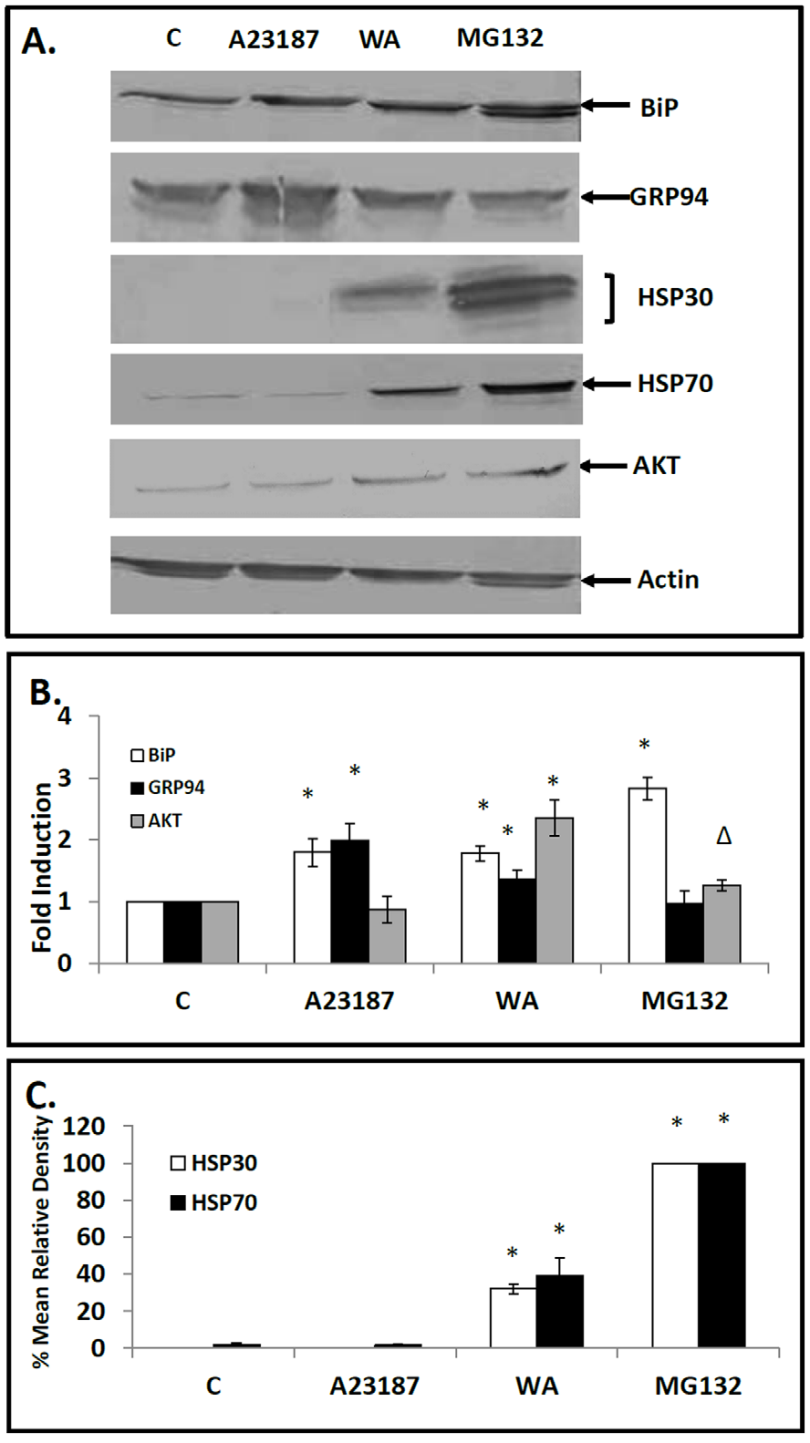
Effect of WA on BiP, GRP94, HSP30 and HSP70 accumulation. A) Cells were treated with 5 µM WA for 16 h, 7 µM A23187, 30 µM MG132 or the appropriate volume of the DMSO vehicle (C) for 24 h at 22°C. Cells were harvested and total protein was isolated. Forty µg of the different protein samples were analyzed by Western blot analysis using anti-BiP, anti-GRP94, anti-HSP30, anti-HSP70, anti-AKT or anti-actin antibodies as described in Material and methods. Image J software was used to perform densitometric analysis of the signal intensity for BiP, GRP94, AKT, HSP30 and HSP70 protein bands of western blot images as described in Materials and methods. The data are expressed for each treatment as a ratio to control levels (for BiP, GRP94 and AKT accumulation) or as percentage of the maximum signal (30 µM MG132 for HSP30 and HSP70 accumulation). The standard error is represented by vertical error bars. The level of significance of the differences between samples was calculated by one-way ANOVA with a Tukey’s post-test. Significant differences between the control cells and treated cells are indicated as * (p<0.05) or Δ (p<0.10). These data are representative of three separate experiments.

**Figure 4:**
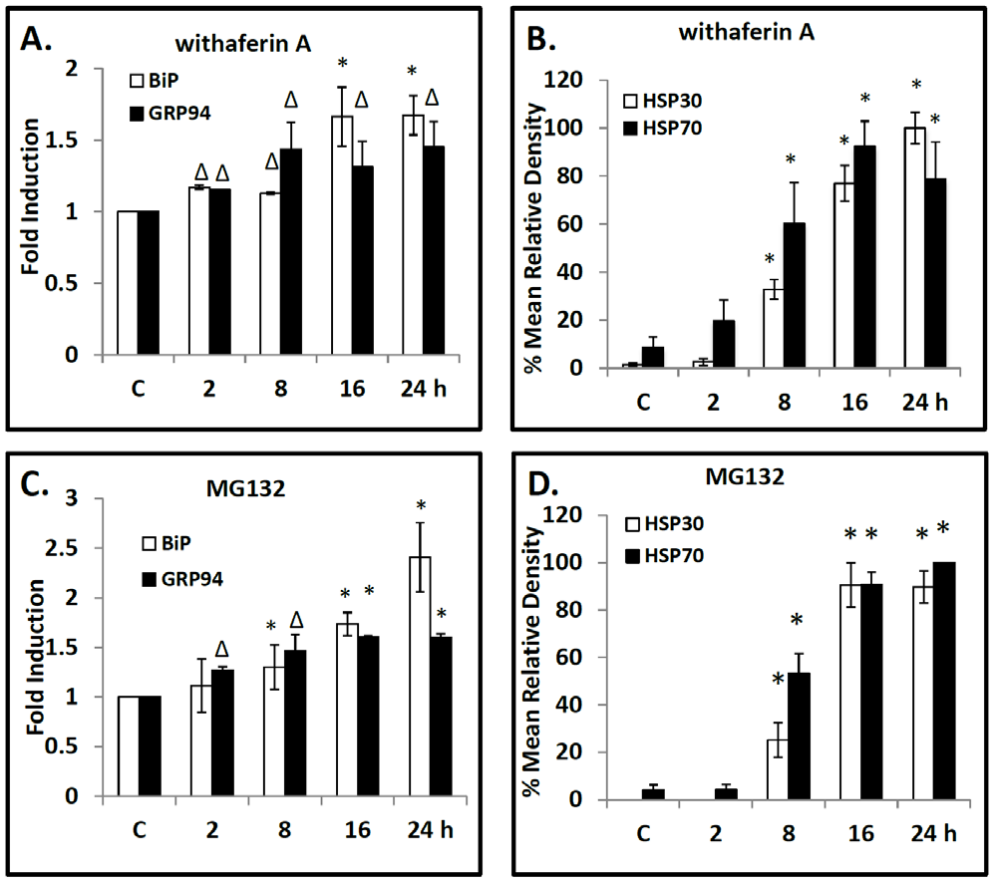
Temporal pattern of WA-induced BiP, GRP94, HSP30 and HSP70 accumulation. Cells were exposed to 5 µM WA (A, B) or 30 µM MG132 (C, D) for time intervals ranging from 2 to 24 h. Cells were harvested and total protein was isolated. The different protein samples were then analyzed by immunoblot analysis. Image J software was used to perform densitometric analysis of the signal intensity for BiP (white), GRP94 (black), HSP30 (white) and HSP70 (black) protein bands of western blot images as described in Materials and methods and in the legend of Figure 3. The data are expressed for each treatment as a ratio to control levels for BiP and GRP94 accumulation (panels A & C) or as a percentage of the maximum band for HSP30 and HSP70 accumulation (panels B & D). Significant differences between the control cells and WA or MG132 treated cells are indicated as * (p<0.05) or Δ (p<0.10). These data are representative of three separate experiments.

WA treatment of cells induced upregulation of BiP, GRP94, HSP70 and HSP30 protein levels (Fig. 3). For example, treatment of cells with 5 µM WA for 16 h resulted in a 2.0 and 1.5-fold increase in BiP and GRP94, respectively, and 36- and 40-fold increases in HSP70 and HSP30, respectively. MG1342 strongly induced BiP, HSP70 and HSP30 while GRP94 levels were not enhanced. As expected the ER stressor, A23187, had no detectable effect on HSP70 or HSP30 but did elevate the relative levels of BiP and GRP94. For comparative purposes, we examined the relative levels of Akt, a protein kinase that is involved in multiple signaling pathways (cell growth, proliferation, survival and metabolism) and is degraded by the ubiquitin proteasome system [56], [57]. While A23187 had no effect on Akt levels compared to control the relative levels of Akt after WA and MG132 treatment increased 2.3 and 1.3 fold, respectively. Additionally, the relative levels of actin were not affected by these treatments. In time course studies of cells treated with 5 µM WA, slightly elevated levels of BiP and GRP94 were observed at 2 h compared to control with relatively higher levels by 16 to 24 h (Fig. 4A). A comparable temporal pattern of ER stress protein induction was determined with cells exposed to the proteasomal inhibitor MG132 (Fig. 4C). A different temporal pattern with respect to HSP70 and HSP30 accumulation was found for cells treated with 5 µM WA with significantly enhanced levels after 8 h of WA treatment and a 20- and 100-fold increase, respectively, by 24 h (Fig. 4B). Again, a similar temporal pattern was observed with the relative levels of HSP70 and HSP30 induced by MG132 (Fig. 4D). Actin levels were not affected by these treatments (data not shown). These experiments reveal that WA treatment can enhance the accumulation of both ER and cytosolic HSPs.

### Mild Heat Shock can Enhance WA-induced HSP Accumulation

**Figure 5:**
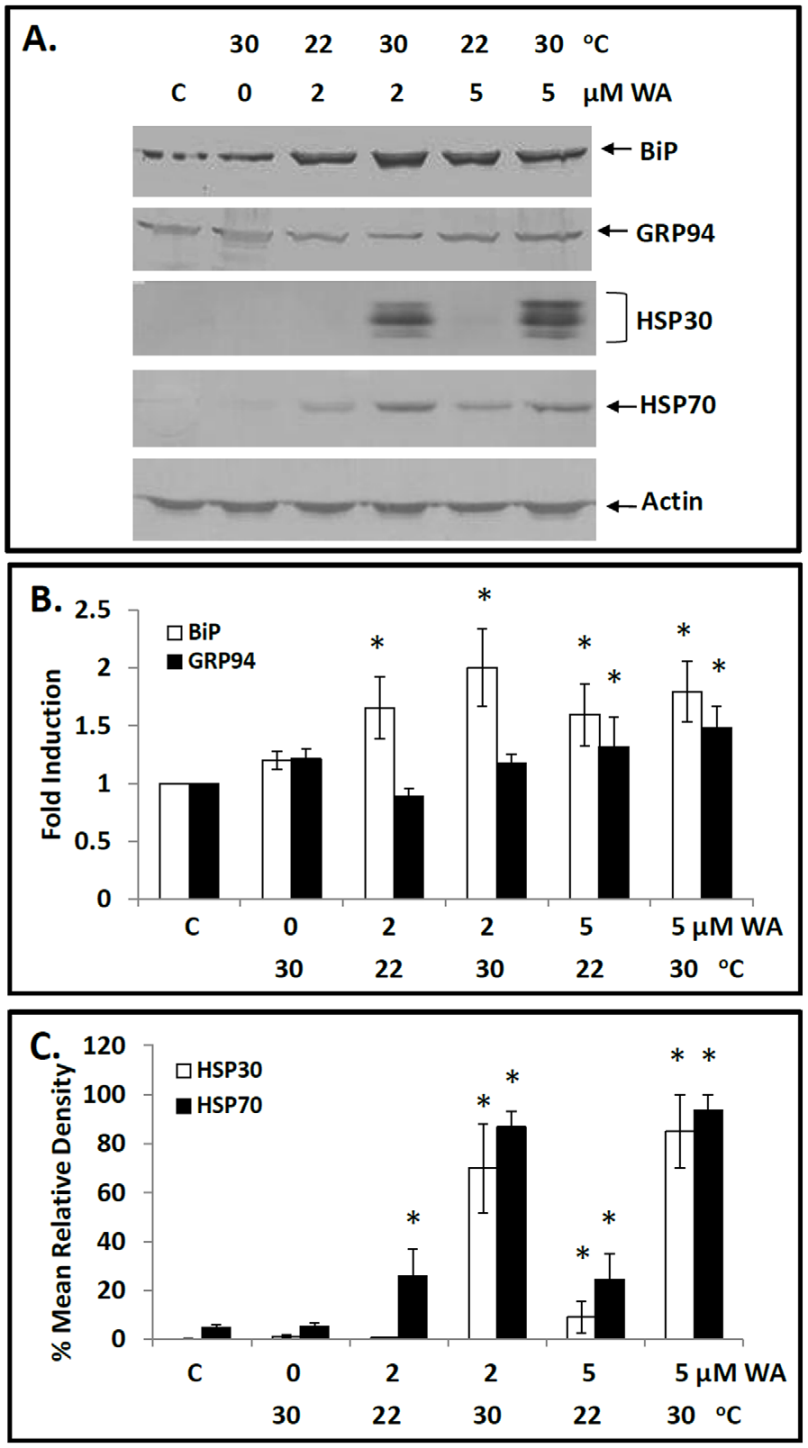
Effect of mild heat shock on WA-induced BiP, GRP94, HSP30 and HSP70 accumulation. A) Cells were exposed to 2 or 5 µM WA at 22°C or 30°C for 8 h. Following treatment, cells were harvested and total protein was subjected to immunoblot analysis. B & C) Image J software was used to perform densitometric analysis of the signal intensity for BiP, GRP94, HSP30 and HSP70 protein bands of western blot images as described in Materials and methods and in the legend for Figure 3. The data are expressed for each treatment as a ratio to control levels (for BiP and GRP94 accumulation) or as percentage of the maximum signal (combined WA and mild heat shock treatment for HSP30 and HSP70 accumulation). Significant differences between the control cells and treated cells are indicated as * (p<0.05). These data are representative of three separate experiments.

Since previous investigations reported that mild heat shock treatments were capable of enhancing chemical stress-induced *hsp* gene expression [18], [48], [54], this phenomenon was examined with WA. Cells exposed to 30°C singly produced no significant increase in BiP or GRP94 accumulation. In contrast, cells exposed to 2 µM WA for 8 h at 22°C showed an increase in BiP accumulation, which was further elevated with the concurrent treatment of cells at 30°C (Fig. 5). Moreover, cells exposed singly to 30°C or treated with 2 µM WA for 8 h at 22°C had a low relative level of HSP70 while HSP30 was not detectable (Fig. 5). In contrast, concurrent treatment of cells with 2 µM WA at 30°C resulted in enhanced levels of HSP70 and HSP30 accumulation that were greater than the sum found with each stress individually. Similar results were obtained with concurrent exposure of cells to 30°C plus 5 µM WA for 8 h compared to cells exposed to mild heat shock or 5 µM WA alone. In this latter experiment, comparable findings were obtained at other treatment times including 4 and 12 h (data not shown). The relative levels of actin were not altered by these different treatments. These results indicate that combined mild heat shock and WA treatment can act synergistically on the accumulation of HSP70 and HSP30.

### Intracellular Localization and Accumulation of BiP and HSP30 in WA Treated Cells

**Figure 6:**
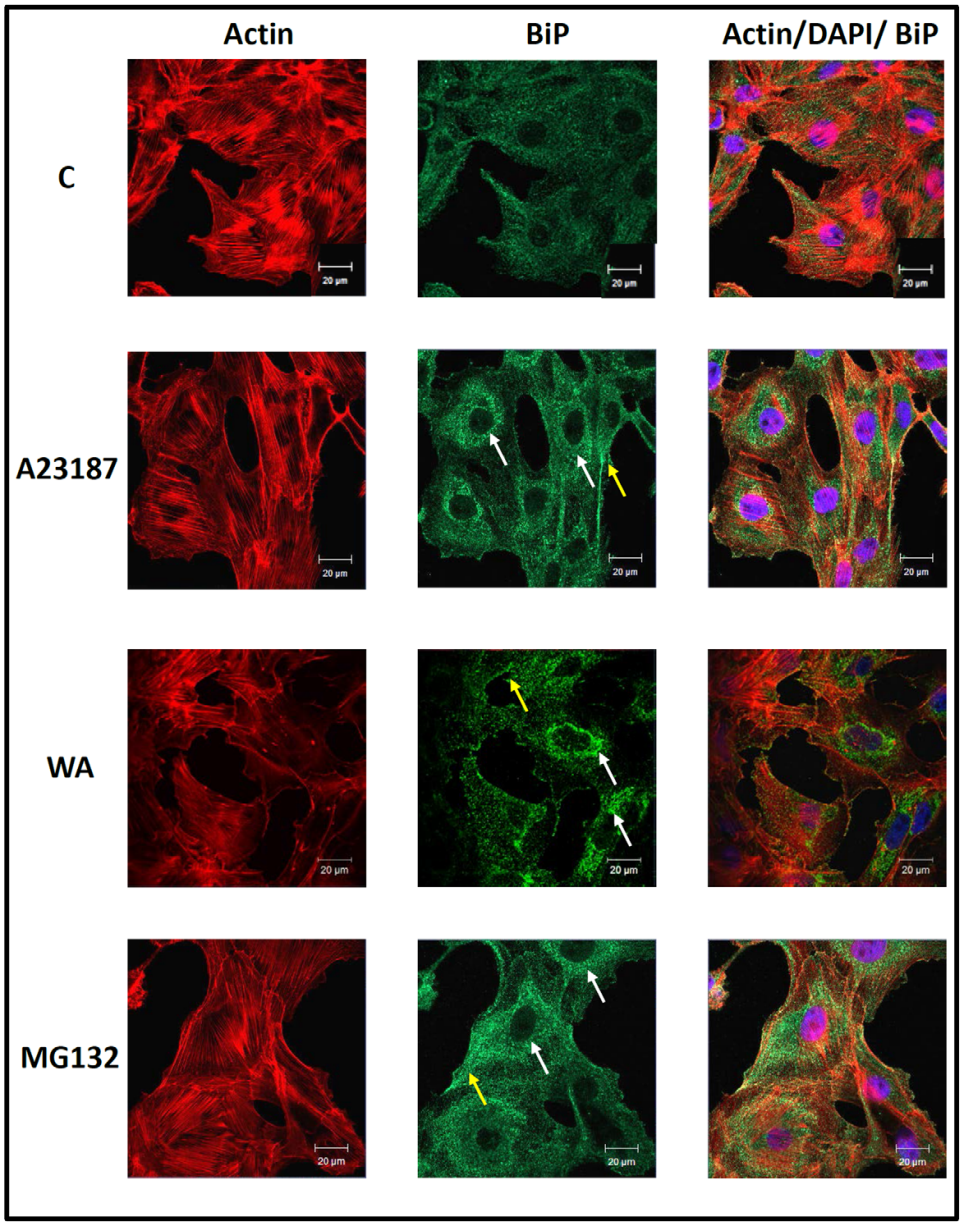
Intracellular localization of BiP in response to A23187, WA and MG132. Cells were grown on glass coverslips and were treated with 5 µM WA for 16 h, 7 µM A23187 or 30 µM MG132 for 24 h at 22°C. BiP was indirectly detected with an anti-BiP antibody and a secondary antibody conjugated to Alexa-488 (green). Actin and nuclei were stained directly with phalloidin conjugated to TRITC (red) and with DAPI (blue), respectively. From left to right the columns display fluorescence detection channels for actin, BiP and merger of actin, DAPI and BiP. The white arrows indicate perinuclear localization while the yellow arrows show the presence of BiP near the cell membrane. The 20-µm white scale bars are indicated at the bottom right section of each panel. These results are representative of 3 different experiments.

**Figure 7:**
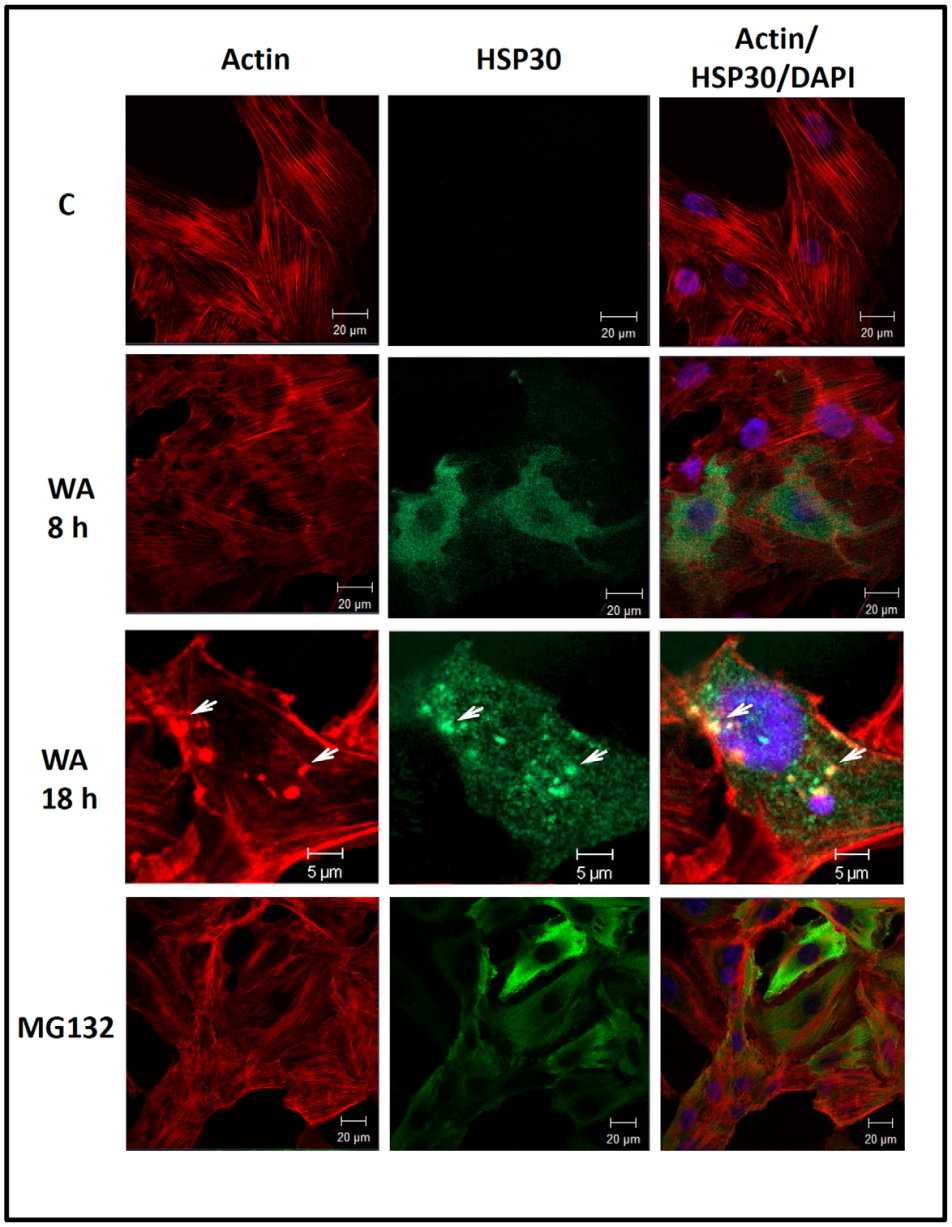
Localization of WA-induced HSP30 accumulation. Cells were cultured on glass coverslips and incubated with 5 µM WA for 8 or 18 h or 30 µM MG132 for 24 h at 22°C. HSP30 was indirectly detected with an anti-HSP30 antibody and a secondary antibody conjugated to Alexa-488 (green). Actin and nuclei were stained directly with phalloidin conjugated to TRITC (red) and with DAPI (blue), respectively. In the 18 h set of images (bottom row), the white arrows indicate examples of structures that display co-localization of both actin and HSP30. The 5 or 20 µM white scale bars are indicated at the bottom right section of each panel. These data are representative of three separate experiments.

The intracellular localization of BiP and HSP30 was examined in cells treated with WA, A23187 and MG132 using immunocytochemistry and laser scanning confocal microscopy (Fig. 6 and 7). Comparable studies with GRP94 or HSP70 were not carried out since their antibodies, which performed appropriately in immunoblot analyses, did not specifically detect GRP94 or HSP70 by immunocytochemistry. A6 cells maintained at 22°C displayed BiP localization diffusely throughout the cell except for the nucleus (Fig. 6). Cells treated with 5 µM WA, 7 µM A23187 or 30 µM MG132 displayed an enhanced accumulation of BiP in the perinuclear region of the cytoplasm in a punctate pattern and occasionally at the periphery of the cell membrane in comparison to control cells. Additionally, in WA-treated cells relatively large BiP staining structures were found at the periphery of the nucleus. Treatment of cells with 5 µM WA for 8 or 18 h resulted in the accumulation of HSP30 in a punctate pattern primarily in the cytoplasm with a small amount in the nucleus (Fig. 7). Examination of the general morphology and actin organization revealed that cells exposed to WA for 8 h displayed an intact cytoskeleton and visible stress fibers, similar to control cells. In contrast, some cells treated for 18 h with WA displayed some F-actin disorganization as well as areas of F-actin aggregation and ruffled edges at the cell periphery. Colocalization of both F-actin and HSP30 was observed in relatively large densely stained structures (Fig. 7; white arrows). Thus, WA-induced BiP accumulation occurred mainly in the perinuclear region while HSP30 accumulated primarily in the cytoplasm with some staining in the nucleus. Furthermore, extended exposure to WA induced a disorganization of the F-actin cytoskeleton as well as the accumulation of larger HSP30 staining structures that co-localized with F-actin.

### WA Treatment can Protect the F-actin Cytoskeleton Against Thermal Damage

**Figure 8:**
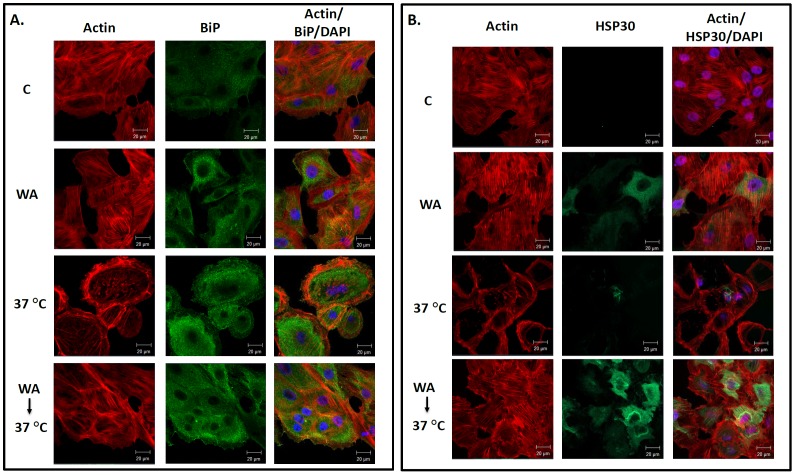
Cytoprotective effects of pretreating cells with WA prior to a thermal challenge. Cells were maintained at 22°C in the presence of the appropriate volume of the DMSO vehicle (C; top row) or exposed to 2 µM WA for 6 h (second row) or a 37°C thermal challenge for 1 h (third row) followed by a 6 h recovery period at 22°C. Additionally, some cells were exposed to 2 µM WA for 6 h followed by a 12 h recovery at 22°C in WA-free media prior to a 37°C heat shock for 1 h with a 6 h recovery at 22°C (bottom row). A) BiP was indirectly detected with an anti-BiP antibody and a secondary antibody conjugated to Alexa-488 (green). Actin and nuclei were stained directly with phalloidin conjugated to TRITC (red) and DAPI (blue), respectively. From left to right the columns display fluorescence detection channels for actin, BiP and merger of actin, DAPI and BiP. The 20 µM white scale bars are indicated at the bottom right section of each panel. B) HSP30 was indirectly detected with an anti-HSP30 antibody and a secondary antibody conjugated to Alexa-488 (green). Actin and nuclei were stained directly with phalloidin conjugated to TRITC (red) and DAPI (blue), respectively. The 20 µM white scale bars are indicated at the bottom right section of each panel. These data are representative of 3 separate experiments.

Previous studies have shown that treatment of cells with agents that can promote the accumulation of HSPs acquire a state of cellular thermotolerance and cytoskeletal thermoresistance [18], [25], [43]. In the present study, cells were exposed to WA treatment prior to a thermal challenge at 37°C to determine whether this compound could confer a cytoprotective effect (Fig. 8). Shifting the incubation temperature from 22°C directly to a 37°C thermal challenge for 1 h resulted in the collapse of the F-actin cytoskeleton. However, pretreatment with 2 µM WA for 6 h plus a 12 h recovery period, which induced BiP (Fig. 8A) and HSP30 (Fig. 8B) accumulation, resulted in maintenance of F-actin cytoskeletal organization and stress fibers after the thermal challenge. Similar results with respect to the acquisition of thermotolerance were obtained in cells treated concurrently with 2 µM WA and 30°C heat shock prior to a 37°C thermal challenge (data not shown). In summary, these experiments indicate that WA treatment of cells that was sufficient to induce the accumulation of HSPs conferred a state of thermal protection since it protected the F-actin cytoskeleton against thermal damage.

## Discussion

The present study has shown that treatment of *Xenopus* cells with 5 µM WA induced an inhibition of proteasome activity and the induction of both ER and cytoplasmic/nuclear molecular chaperones in a time-dependent manner. WA-induced inhibition of proteasomal activity was indicated by an increase in ubiquitinated protein and a decrease in proteasomal CT-like activity. These results are similar to findings with human prostate cancer cells in which treatment with 5 to 20 µM WA resulted in an increase in ubiquitinated protein in a dose-dependent manner and a 50% inhibition in chymotrypsin-like activity after treatment with 20 µM WA for 16 h [3]. In this latter study, it was suggested that WA-induced proteasomal inhibition resulted from a direct interaction between WA and the β5 subunit of the 20S proteasome, which possesses CT-like activity. It is possible that WA may have a similar effect on the β5 subunit in *Xenopus* cells.

Activation of the unfolded protein response by 5 µM WA was suggested by the accumulation of *bip* mRNA and BiP and GRP94 protein. These results are in agreement with a recent study by Choi et al. [50] who determined that treatment of mammalian Caki cells with 2–6 µM WA induced ER stress as indicated by the enhanced accumulation of *bip/grp78* mRNA and encoded protein. While the mechanism responsible for WA and MG132 induction of the ER chaperones in *Xenopus* cells is not known, it is possible that WA may cause an increase in unfolded and/or misfolded proteins in the ER lumen resulting from the repression of proteasomal activity. As mentioned previously an increase in unfolded protein results in enhanced accumulation of BiP and GRP94 in order to inhibit the production of protein aggregates and maintain client protein in a competent state for subsequent folding, oligomerization and translocation [7]. A WA-induced increase in the relative levels of Akt, a protein kinase involved in multiple cellular pathways, may reflect generalized proteasomal inhibition since it is known that Akt degradation involves the ubiquitin proteasome system [56], [57]. This latter finding supports the theory that generalized WA-induced proteasomal inhibition may trigger the unfolded protein response. Furthermore, it is also possible that WA-induced proteasomal inhibition may slightly increase the relative levels of HSPs after they are induced since they cannot be degraded. In these experiments, the relative levels of actin were not altered. It is possible that actin’s relatively long half-life and abundance in the cell may mask an increase in its relative levels by WA-induced proteasomal inhibition.

The accumulation of HSP30 and HSP70 as well as their mRNAs in WA-treated cells is likely the result of an activation of the heat shock response. Previous studies found that WA bound to cysteine residues in the C-terminus of HSP90 and inhibited its chaperone activity by disrupting its ability to bind to client proteins [20]. Recently, it was determined that the α, β-unsaturated carbonyl motif of WA reacted with proteins by forming adducts with thiol groups in cysteine residues and that this motif was necessary but not sufficient for heat shock activation of green fluorescent protein controlled by a minimal consensus HSE-containing promoter in a reporter cell line [21]. Since monomeric inactive HSF1 is normally bound to HSP90, binding of WA to HSP90 might disrupt this complex allowing HSF1 to trimerize thus activating the expression of *hsp* genes. Additionally or alternatively, WA-induced proteasome inhibition might cause an increase in unfolded or damaged proteins that could activate the heat shock response as suggested for other stressors including heat shock and MG132 [18], [48], [49], [58], [59]. This latter model is supported by our finding that the relative levels of Akt increased in response to WA treatment compared to control.

In the present study, concurrent treatment of cells with both mild heat shock and WA induced elevated levels of HSP70 and HSP30 accumulation. In fact, the relative levels of HSP70 and HSP30 induced by the concurrent stresses were greater than the sum of the levels found with each stress individually. This phenomenon was not observed with BiP or GRP94 accumulation. The mechanism responsible for elevated levels of HSP70 and HSP30 induced by combined WA and mild heat shock is not clear. The possible WA-induced dissociation of the HSF1/HSP90 complex and/or increased in damaged protein coupled with a generalized protein unfolding caused by mild heat shock temperatures may enhance the activation of HSF1 leading to *hsp* gene expression.

Immunocytochemistry was employed to determine the localization of BiP and HSP30 in cells treated with WA. Cells treated with WA, MG132 or A23187 had an enhanced accumulation of BiP in a punctate pattern in the perinuclear region and occasionally at the periphery of the cell membrane. The enhanced accumulation and localization pattern of BiP in A6 cells is indicative of an unfolded protein response. For example treatment of cells with ER stressors such as thapsigargin, a Ca2+-ATPase inhibitor, or tunicamycin were reported to upregulate BiP in a punctate pattern with coalescence of BiP in the perinuclear region of mammalian cells [60], [61]. A similar response was also reported with proteasome inhibitors, MG132 and/or lactacystin in mouse pancreatic β-cell cytoplasm and dog MDCK cells [14], [60], [62]. Additional immunocytochemical analysis determined that A6 cells exposed to 5 µM WA for 8 h accumulated HSP30 mainly in the cytoplasm in a granular pattern with a lesser amount of HSP30 staining in the nucleus. Furthermore, cells treated with WA for 8 h did not display a disruption in the F-actin cytoskeleton, which has been employed as a positive indicator of cellular health [63], [64]. The punctate pattern of HSP30 accumulation may reflect the stress-induced formation of HSP30 multimeric structures that are associated with its cellular function in the prevention of toxic aggregates [25], [29], [30], [65]. In contrast, treatment of cells with WA for 18 h produced relatively large HSP30 staining foci. Similar large HSP30 staining structures were reported in A6 cells exposed to proteasomal inhibitors [18]. Although the identity of these HSP30 staining foci is unknown, it is possible that they are inclusion bodies containing HSP30 bound to unfolded proteins given the molecular chaperone function of HSP30 [18], [44]. In studies with mammalian cells, it was determined that proteasome inhibitors can significantly increase the formation of cytosolic aggresomes, which are proteinaceous inclusion bodies that form as a general cellular response to aggregated protein [66], [67]. In some WA-treated A6 cells a co-localization of HSP30 with actin was observed which is in accord with the potential role of sHSPs in stabilization of the actin cytoskeleton [29], [30], [67]. Also incubation of A6 cells with WA for 18 h resulted in a general disorganization of the F-actin cytoskeleton, F-actin aggregation and the presence of ruffled membranes. Studies in mammalian systems suggested that WA-induced F-actin aggregation may involve the interaction of WA with the intermediate filament protein vimentin and annexin II, which has basal F-actin cross-linking activity [68], [69]. The mechanism responsible for WA-induced cell membrane ruffling is not well understood. In general, ruffling is the formation of a motile cell surface that contains a meshwork of newly polymerized actin filaments under the plasma membrane and may indicate global instability of cellular adhesion [68].

Finally, the present study determined for the first time that WA induced a state of cellular thermotolerance which permitted cells to maintain their F-actin cytoskeletal organization after a subsequent cytotoxic thermal challenge at 37°C. In support of these findings, the induction of HSP accumulation and acquisition of thermotolerance by the proteasome inhibitor, MG132, has been described in yeast, *Xenopus* and mammalian cultured cells [7], [18], [70], [71]. The WA-induced accumulation of BiP and GRP94 likely inhibits the formation of toxic aggregates in the ER resulting from a potentially lethal heat shock while HSP70 and HSP30 have an analogous function in the cytoplasm and nucleus.

In conclusion, this study has shown that withaferin A can induce proteasomal inhibition, ER and cytoplasmic/nuclear HSP accumulation and confer F-actin cytoskeletal thermoresistance. Given these results, it is tempting to speculate that the upregulation of ER and cytoplasmic/nuclear chaperones by WA may contribute to its potential therapeutic roles in the treatment of various human diseases. Finally, the finding that a mild heat shock greatly enhanced the effectiveness of WA in activating the heat shock response may permit the use of lower concentrations of WA in combination with heat in clinical applications.
